# Examining End-of-Life Case Management: Systematic Review

**DOI:** 10.1155/2014/651681

**Published:** 2014-06-04

**Authors:** Roger E. Thomas, Donna M. Wilson, Stephen Birch, Boris Woytowich

**Affiliations:** ^1^Department of Family Medicine, University of Calgary, Calgary, AB, Canada T2N 4N1; ^2^Faculty of Nursing, University of Alberta, Edmonton, AB, Canada T6G 1C9; ^3^Centre for Health Economics and Policy Analysis, McMaster University, Hamilton, ON, Canada L8S 4K1

## Abstract

Case management was initiated in the 1970s to reduce care discontinuity. A literature review focused on end-of-life (EOL) case management identified 17 research articles, with content analysis revealing two themes: (a) seeking to determine or establish the value of EOL case management and (b) identifying ways to improve EOL case management. The evidence, although limited, suggests that EOL case management is helpful to dying individuals and their families. Research is needed to more clearly illustrate its usefulness or outcomes and the extent of need for it and actual availability. Among other benefits, EOL case management may help reduce hospital utilization, a major concern with the high cost of hospital-based care and the increased desire for home-based EOL care.

## 1. Examining End-of-Life Case Management

### 1.1. Findings of a Systematic Review of Published Research

The Case Management Society of America [1] defines case management as “a collaborative process of assessment, planning, facilitation, care coordination, evaluation, and advocacy for options and services to meet an individual's and family's comprehensive health needs through communication and available resources to promote quality, cost-effective outcomes” (paragraph 1). Case management was introduced in the United States during the 1970s and then in Canada, New Zealand, Australia, Ireland, the United Kingdom, and much of continental Europe [2–4]. It was primarily initiated out of growing awareness of and concerns over discontinuity of care [5–7]. Early reports indicated that it was used to reduce or prevent care discontinuity for individuals suffering from long-term serious conditions such as mental illnesses, diabetes, heart failure, and chronic substance abuse [7–13]. People with complex or multiple health issues, such as the frail elderly, were also considered prime candidates for case management [7–14]. Frail elderly persons are typically very old, with supportive or palliative care often a more appropriate care modality for them rather than aggressive cure-oriented diagnostic tests and treatments [15].

In Canada, one of every five decedents is 85 years of age or older at the time of death [16]. Regardless of whether dying people are very old and thus likely to be frail elderly persons or not, terminally ill and dying people often have multiple health issues and care needs [17]. Case management could, therefore, potentially be helpful to them. Unfortunately, no systematic review of EOL case management has yet been published. We undertook a systematic literature review with the aim of understanding what research evidence exists on EOL case management.

### 1.2. Systematic Search and Review Method

After consulting with a university librarian, we searched EMBASE, MEDLINE, CINAHL, AHMED, ERIC, CancerLit, HealthStar, PsychLit/Psychinfo, the Cochrane Library (including the Cochrane Controlled Trials Register and Library of Systematic Reviews), and sociological abstracts for all EOL case management research articles published in the years 1989 (the year of the first international conference on EOL care for seniors [18]) through 2012. As the terms “case management” and “care management” are often used interchangeably [6], the search terms were “case manage∗” and “care manage∗” to retrieve all forms of case/care management and case/care manager. We combined the results with the MESH/keywords: “terminal care,” “palliative care,” “hospice,” “dying,” or “end of life.” The search was then limited to English language research articles, with just over 400 abstracts identified. Two researchers independently assessed these abstracts, and 19 articles that described research on one or more aspects of EOL case management were identified. After these 19 papers were read in full, five articles were excluded as they did not provide information on the research methods that were used to gather and/or analyze data.

Although this search identified some general reviews of case management [6, 12, 19–21], these were excluded as none focused on EOL case management. Also rejected for review were around 380 discussion or opinion articles on EOL case management. These nonresearch papers represent evidence, however, of considerable interest in the topic of EOL case management.

The reference lists of the 14 articles were then scanned, and two additional eligible articles were thus identified. In addition, a manual search undertaken of three journals that could be expected to contain research articles on EOL case management (i.e., Journal of Palliative Care, Journal of Palliative Medicine, and Journal of Case Management) identified one additional article, for a total of 17.

After this search and selection process, two reviewers independently assessed each article to tabulate key study design and outcome data. These findings were compared and consolidated into a table. The two reviewers then worked together in performing a content analysis to identify and categorize findings and then group the categories into themes; a ground-up qualitative analysis process was suggested by Higgins and Green [22] and also Wells et al. [23]. Through this process, two themes became evident: (a) seeking to determine or establish the value of EOL case management and (b) identifying ways of improving EOL case management.

Risk of bias of the RCTs was assessed using the methods recommended in the Cochrane Collaboration* Handbook* [24], cohort studies with the Newcastle-Ottawa scale [25], and nonrandomized studies that were not cohort studies with CASP tool [26].

## 2. Results

### 2.1. Risk of Bias

We identified five RCTs (Table 1), two of which [27, 28] were at low risk of bias for all six elements of risk of bias assessed, and the others had mixtures of low, unclear, and high risks of bias. We identified six cohort studies (Table 2), five of which [29–33] were at low risk of bias. We identified four other nonrandomized designs (Table 3), two of which [34, 35] were at low risk of bias. We also identified a case study of a single individual [36].

### 2.2. Focus of Care

Thirteen studies were of palliative care patients [29–40, 43] and four were about frail patients [27, 28, 41, 42].

### 2.3. Analysis of Studies

We identified two key themes in the literature: seeking to determine or establish the value of EOL case management and identifying ways to improve EOL case management. The characteristics of the studies are presented in Table 4.

#### 2.3.1. Theme 1: Seeking to Determine or Establish the Value of EOL Case Management

Fourteen of the 17 reviewed studies were designed to assess or examine and thus determine or establish the value of EOL case management. Six of these studies focused on hospital utilization with the intention of determining an economic value to EOL case management. The eight other studies focused on additional value considerations.

*Subtheme 1a: Hospital Utilization*. As indicated, six studies focused on hospital utilization, with EOL case management researched as to whether it could reduce hospital admissions and/or hospital stays at or near the end of life and thus reduce healthcare costs [28–30, 33, 37, 42]. All of these studies were undertaken in the United States. The findings from these studies are contradictory; four studies found economic benefit while two did not.

Among those reporting economic benefit, Naylor et al. [28] found that seniors who received EOL case management for four weeks following hospital discharge were less likely to be hospitalized in the subsequent six month study period as compared to a control group. Healthcare costs for case-managed clients were approximately half those of the control group. Back et al. [29] similarly found among persons dying of cancer that palliative case management for 60 or more days before death resulted in a lower use of acute care hospital resources and thus healthcare costs, compared to those without palliative case management. Seow et al.'s [30] study also found that cancer patients who received EOL case management were much less likely to be hospitalized than the control patients. Elwyn et al.'s [42] qualitative study revealed that the reduction in hospital utilization that occurred with EOL case management was likely a result of terminally ill people having a higher quality of life and with this outcome leading to a reduced need for hospital-based EOL care.

In contrast, Twyman and Libbus [33] found no difference in hospital use between persons diagnosed with AIDS who had or had not received EOL case management over the last six months of life. Furthermore, Long and Marshall's [37] study found that case-managed elderly persons were more likely to be hospitalized and to use other health services during the last month of life as compared to those who did not receive EOL case management. Although research methodology differences and methodological concerns may explain the conflicting hospital utilization and cost findings across these six studies, Long and Marshall [37] and Twyman and Libbus [33] indicated that the nurse case managers had acted as client advocates and so had assisted their clients in obtaining needed health services, including hospital-based care. As such, these six studies help to reveal a number of potential intended outcomes (such as coordinating services to reduce the need for hospital-based care) and also unintended or secondary outcomes or consequences (such as identifying service gaps, addressing unmet needs for care, and a higher quality of life) of EOL case management.

*Subtheme 1b*:* Additional Value Considerations*. While six studies focused on hospital utilization with the intention of determining if EOL case management had economic value, eight others focused on additional value considerations [27, 31, 32, 38–41, 43]. These eight studies varied considerably in aim, design and research methods, and findings. Each is briefly outlined below, with additional information presented in the table.

One study used both qualitative and quantitative methods to measure the impact of the withdrawal of EOL case management services from noninstitutionalized seniors and their family caregivers in Hawaii [41]. The authors found a larger than expected number of deaths in the first six months following program cessation, with the surviving ex-clients indicating that the program had been critical for their support and safety. Half of all family caregivers reported that their own physical health had deteriorated and their emotional fatigue increased following program cessation. As such, client and family benefits from EOL case management were identified.

Additional potential benefits of EOL case management were identified in other studies. One study involved a preliminary program evaluation conducted relatively soon after the implementation of case management services for palliative/dying clients [38]. These clients were a new type within an existing case management program. The researchers found that palliative-educated case managers were able to identify and address patient distress, they had a good working relationship with their clients' physicians, and both client and family satisfaction improved under their case management. Head et al. [31] also found a reduction in symptom distress among dying persons and increased client satisfaction with care one month after the onset of EOL case management. Similarly, Pfeifer et al. [40] studied the added value of EOL case management and found that case-managed clients were very positive about the case management help they received, which they described as good advice and direction. Finally, Spettell et al. [32] found that hospice use increased after EOL case managers were hired to work within a U.S. palliative care program. This was the intended outcome, as not only did the patient care focus appropriately shift from curative care to palliative care, but also the terminally ill and dying persons began to receive high quality EOL care in hospices.

Two randomized controlled trials (RCT) of EOL case management also revealed additional benefits. One assessed the quality of life for case-managed home care clients dying of AIDS [39]. This study found that although quality of life scores declined rapidly for both the case-managed and control clients, the case-managed client scores were higher. As such, case-managed clients were determined to have a higher quality of life when dying. The other RCT assessed the effect of EOL case management on persons suffering from end-stage lung or heart failure [27]. A comprehensive impact assessment was conducted, with EOL case-managed clients having fewer symptoms, less symptom distress, more vitality, better physical health, higher self-rated health, and significantly better self-care management, awareness of resources, and legal preparation for the end of life than control group subjects. As such, this study and the others indicated a wide range of potential benefits from EOL case management.

However, one program evaluation study identified a number of concerns with a new program of EOL case management provided through a district coordinating service in England [43]. Minimal contact was found between the clients and case managers, despite the expectation of a visit from the case manager soon after hospital discharge and then ongoing contact until death. Of the 199 clients studied, 21% were never visited or telephoned at home, 62% were visited at home, and 71% were contacted at home by telephone. Just over half (51%) of the telephone calls lasted five minutes or less, and a small proportion lasted one hour or more. One-third of the contacted family members reported the issue that they did not know how to get their family member the EOL care they required and one-third felt that their family member's terminal care was not well coordinated. These findings for these case-managed clients were similar to those of matched clients who did not receive any case management services. Another key finding was that 59% of surveyed healthcare workers in the region had not heard of this new service, although most (87%) thought it could be beneficial. Unfortunately, the paper did not highlight why these problems were present.

#### 2.3.2. Theme 2: Identifying Ways of Improving EOL Case Management

As indicated, three of the 17 reviewed studies focused on improving EOL case management. The first chronologically was a study in Ontario, Canada, where EOL case management was said to be well established [35]. This study found that EOL case managers were challenged by a wide range of service gaps that impacted their clients and client families. These service gaps included an inadequate number of services for the family, few palliative care specialists, and inequalities in both the amount and quality of home care services across the study region. Another issue was case managers not being notified of new clients, with these new clients then not having their needs quickly identified or addressed. Recommendations for changes to improve EOL case management were made, although most were oriented to increasing the supply of EOL services in the community.

A study by Spencer and Battye [34] sought to determine if children dying of cancer in England had received high quality palliative case management. They found many different case management approaches in use. This diversity was not unexpected, however, as much of the care of dying children in England takes place at home, and care circumstances vary considerably across children, families, and regions of the country. Regardless of the different case management approaches, multiagency collaboration and service delivery were found to be common, with the case manager or management team having an important role in arranging and coordinating services across agencies. The authors identified some needed improvements, however, including more communication and better liaison between all care professionals involved in a case, and clearer roles for the case managers or case management teams.

The third and most recent study was conducted in the US after an EOL case management service was started within a managed care organization [36]. The case management team consisted of a nurse and a social worker, both with palliative care specialization. The study revealed that EOL case-managed clients received patient-centered care, and this care was cost effective. The EOL case managers were considered particularly useful when hospice care was not available in the client's community. As such, this study suggests one way of ensuring an effective EOL case management service is to situate it within a larger well established program.

## 3. Discussion

The limited research evidence to date, with only 17 studies focused on EOL case management, suggests that it can improve client and family outcomes, such as satisfaction with care and quality of life while also reducing hospital utilization. However, few research studies have clearly and repeatedly quantified these or other potential outcomes of EOL case management. Unfortunately, this lack of research is not unexpected, as many authors have indicated that case management research is needed [2, 8, 10–12, 20, 21, 44–51].

In the case of EOL case management, the lack of conclusive research evidence could be a result of difficulties in studying this specific service in isolation of other services [52]. However, it is surprising that EOL case management has not been researched more often, as this service may be a standard component of many palliative care programs [17, 28, 30, 33, 35, 40, 53, 54]1. However, many dying people do not receive their EOL care through established palliative care programs [55], and this care circumstance may explain why EOL case management appears to be overlooked as an essential service of considerable possible benefit to most if not all terminally ill persons and their families.

Much descriptive, evaluative, and other research is needed on EOL case management. Surveys of hospitals, hospice/palliative care programs, and home care agencies should establish the existence or extent of EOL case management, as well as the sociodemographic and other characteristics of case-managed clients and their families. Similar descriptive information should be collected on case managers, in part, to determine if nurses are the most common case managers and if most EOL case managers have advanced palliative care education or practical training. As some case managers are nurse practitioners [28], surveys of knowledge and skill requirements for successful case management would provide additional useful information.

Information is also needed on the roles or duties that case managers are expected to fulfill and if or how they are hampered in assisting their clients. The English study that found minimal contact between case managers and clients [43] could, for instance, illustrate a workload that is too high for any case manager to accomplish. The additional finding in that study that 59% of surveyed healthcare workers in the region had not heard of this new service also suggests inadequate service planning. Williams' [35] finding that case managers were challenged by a wide range of community service gaps and that they were not notified of new clients similarly shows how EOL case managers may be hampered in their effectiveness.

Outcomes research is particularly needed, however, as the research literature suggests many possible individual, family, economic, and other benefits of EOL case management. This research should be considered a priority as case management could become more important in the years ahead with a growing number of decedents now that the large baby boom generation has begun to reach old age and because of an expected increase in home-based dying [13, 38, 56]. Home deaths are already becoming more common in Canada and many other countries [16] as the home is now a preferred EOL location for many terminally ill persons and their families [13, 38, 56]. Furthermore, as ill persons increasingly receive their diagnostic tests and treatments on an ambulatory or outpatient basis, discontinuity of care could remain a problem for dying persons and their families.

Although this review found that economic outcomes have been a research focus, which is understandable since EOL case management is intended in some areas to reduce EOL hospital utilization and thus healthcare costs, and case management could be a costly service to implement, future research should focus on the “good” death [57, 58]. Case-managed clients may be more likely to die a good death if care discontinuity issues are eased or prevented by a case manager [17, 57, 58].

In short, the expected or anticipated outcomes of EOL case management need to be clearly outlined and quantified to establish the value of EOL case management. Once this value is established, issues for improvement and ways to advance the effectiveness of EOL case management will be of prime importance. All such efforts need to take into consideration growing title diversity, since “care coordinators,” “patient navigators,” “care coaches,” and “patient advocates” are also used to identify case managers [5]. Moreover, considerable role diversity could be occurring within and across countries, providing another topic for research.

## 4. Conclusion

A review of the published research literature on EOL case management, although limited in scope and depth, indicates that it has much potential for helping terminally ill and dying persons and their families. Some of this benefit may be through reducing the need for hospital-based care and thus reducing terminal healthcare costs. Although this body of evidence does not clearly demonstrate that EOL case management is an essential or a highly effective care modality, such as for reducing hospital utilization or ensuring a “good” death, the available evidence and anecdotal information on the need for and apparent utility of case management mandates further research. Arguably, the most important outcomes of EOL case management are an improved quality of life while dying and assistance to enable “good” deaths. In short, this systematic review has demonstrated that EOL case management is an underresearched but possibly very important service that is needed now as well as in the future.

## Figures and Tables

**Table 1:** Risk of bias assessments of RCTs.

Type of risk of bias	Risk of bias: authors' judgement	Support for judgement
Aiken et al. (2006) [27]		
Random sequence generation (selection bias)	LOW	“Randomization was carried out within diagnosis, in blocks of 30 patients (15 Phoenix Care, 15 control) by a member of the project administration staff. Sealed envelopes, color-coded by diagnosis and containing the assignment to condition, were shuffled and assigned to participants in order of shuffling.”
Allocation concealment (selection bias)	LOW	“Randomization was carried out within diagnosis, in blocks of 30 patients (15 Phoenix Care, 15 control) by a member of the project administration staff. Sealed envelopes, color-coded by diagnosis and containing the assignment to condition, were shuffled and assigned to participants in order of shuffling.”
Blinding of outcome assessment (detection bias)	LOW	“Every 3 months all participants received a 30- to 45-minute telephone interview by a professional interviewing firm; interviewers were blind to condition and diagnosis.”
Incomplete outcome data (attrition bias)	LOW	“At the end of data collection 44% of the PhoenixCare participants and 25% of the control patients were still participating…percentages for PhoenixCare versus controls, respectively were 16% versus 13%, death; 12% versus 13%, hospice; …6% of PhoenixCare declined and 11% controls declined to continue participation, another 10% and 14% respectively, disqualified by leaving their MCO. [Managed Care Organization] “Only one condition by retention interaction was detected that signaled differential attrition, that for having been given sufficient information and education to manage illness at home, *P* < 0.05.”
Selective reporting (reporting bias)	LOW	No selective reporting

Long and Marshall (1999) [37]		
Random sequence generation (selection bias)	UNCLEAR	“For a randomized trial of ambulatory case management, 317 enrollees in the Kaiser Permanente Medical Care Program, Ohio who were 75 years and over, had severe functional disability, or had excessive hospital or emergency department (ED) use were randomly assigned to a Regular Care Group or a Case Managed Group.”
Allocation concealment (selection bias)	UNCLEAR	No statement
Blinding of outcome assessment (detection bias)	HIGH	“Case managers became integral members of the care team, which included the client's personal physician and the physician advisor, who developed the initial care plan for each client. The case managers were responsible for making periodic home visits, reporting back to the care team, and revising care plans as necessary. While case managers made at least one home visit every 6 months, weekly visits to some clients were not uncommon. In addition to this, the case manager scheduled medical appointments, accompanied patients on these appointments and arranged for nonmedical services such as respite care, meals-on-wheels, nursing home placement, Medicaid eligibility, and transport to and from the physician.”
Incomplete outcome data (attrition bias)	LOW	This was a study of care in the last month of life. “the two groups of deceased are statistically comparable to such an extent as to suggest that statistical benefits of the initial random assignment persisted even in death.”
Selective reporting (reporting bias)	LOW	No selective reporting
Other bias	LOW	No other biases ascertained

Meier et al. (2004) [38]		
Random sequence generation (selection bias)	UNCLEAR	“Care Coordinator nurses were randomly assigned to provide either usual case management (4 nurses) or the palliative care enhanced intervention (5 nurses).”
Allocation concealment (selection bias)	UNCLEAR	No statement
Blinding of outcome assessment (detection bias)	UNCLEAR	No statement
Incomplete outcome data (attrition bias)	NOT APPLICABLE	Program description, no quantitative results
Selective reporting (reporting bias)	NOT APPLICABLE	Program description, no quantitative results
Other bias	NOT APPLICABLE	Program description, no quantitative results

Naylor et al. (1999) [28]		
Random sequence generation (selection bias)	LOW	“assigned to study group using a computer generated algorithm”
Allocation concealment (selection bias)	LOW	“RAs, who were responsible for enrolling patients in the study were blinded to study groups and hypotheses”
Blinding of outcome assessment (detection bias)	LOW	“Outcome data were collected by RAs blinded to study groups and hypotheses”
Incomplete outcome data (attrition bias)	LOW	Attrition rate (including deaths) in intervention group 30%, in control group 26%. “For patients who did not complete the entire 24-week postindex hospitalization study period (death or withdrawal), data collected between randomization and withdrawal were used in the analyses, performed according to the intention-to-treat principle,” “The 262 patients who completed the study and the 101 persons in the attrition group did not significantly differ in sociodemographic variables and severity of illness measures (e.g., number of comorbid conditions).”
Selective reporting (reporting bias)	LOW	No selective reporting
Other bias	LOW	No other biases ascertained

Nickel et al. (1996) [39]		
Random sequence generation (selection bias)	UNCLEAR	“Participants were stratified by agency and randomly assigned to the case-managed or usual-care groups in precoded blocks of two”
Allocation concealment (selection bias)	UNCLEAR	No statement
Blinding of outcome assessment (detection bias)	HIGH	“The schedule of data collection included administration of the ADL and IADL scales by agency nurses at intervals of at least 1week and monthly administration of the QWB by the data collector.” “Since nurses at the seven participating agencies could be assigned to both experimental and control patients, diffusion of intervention practices was of concern.” “Both the case managed and usual-care groups received monthly home visits by project staff for assessment of quality of life outcomes.”
Incomplete outcome data (attrition bias)	HIGH	“Scores for deceased subjects were entered as zero at the monthly time points following occurrence of death. Missing QWB scores in living patients were imputed through linear regression with predicted scores based on individual-specific ADL and IADL scores at time points proximal to the missing QWB times. For time points with ADL/IADL scores also missing, values were imputed through maximum likelihood estimates incorporated within the BMDP program”.
Selective reporting (reporting bias)	LOW	No selective reporting
Other bias	LOW	No other biases ascertained

Risk of bias was assessed according to the methods recommended in: Higgins JPT, Green S (editors). *Cochrane Handbook for Systematic Reviews of Interventions *Version 5.1.0 [updated March 2011]. The Cochrane Collaboration, 2011. Available from http://handbook.cochrane.org/ [22].

**Table 2:** Assessments of risk of bias in included studies, according to Newcastle-Ottawa scale.

Study	Selection				Comparability		Outcome		
Author and date	Representativeness of exposed cohort	Selection of nonexposed cohort	Assessment of outcome	Ascertainment of exposure	Demonstration outcome of interest not present in study start	Study controls for age	Study controls for any additional factor	Follow-up long enough for outcomes to occur	Adequacy of follow-up of cohorts
Back et al. (2005) [29]	∗	∗	∗	∗	∗		∗	∗	∗
Head et al. (2010) [31]	∗	∗	∗	∗	∗		∗	∗	a
Pfeifer et al. (2006) [40]			∗	∗			∗	∗	∗
Seow et al. (2008) [30]	∗	∗	∗	∗		∗	∗	∗	∗
Spettell et al. (2009) [32]	∗	∗	∗	∗	∗	∗	∗	∗	∗
Twyman and Libbus (1994) [33]	∗	∗	∗	∗	∗	∗	∗	∗	∗

^a^Only 35 of the 68 patients consented to participate in the research project.

Case study: Head and Cantrell (2009), case study of one individual [36].

**Table 3:** Assessments of risk of bias in included qualitative studies, according to the Critical Appraisal Skills Program (CASP) tool for qualitative atudies.

Study	Screening questions		Detailed questions								Risk of bias
	(Yes/cannot tell/no)		(Yes/cannot tell/no)							
Author and date	Clear statement of the aims of the research	Is a qualitative methodology appropriate	Appropriate research design	Recruitment strategy appropriate	Data collected so that research issue are addressed	Relationship between researcher and participant adequately considered	Ethical issues taken into consideration	Data analysis rigorous	Clear statement of findings	How valuable is the research?	
Browne and Braun (2001) [41]	Yes	Yes	Yes	Yes	Cannot tell	Yes	Cannot tell	No	Yes	Low	High
Elwyn et al. (2008) [42]	Yes	Yes	Yes	Yes	Cannot tell	Yes	Cannot tell	No	Yes	Low	High
Spencer and Battye (2001) [34]	Yes	Yes	Yes	Yes	Yes	Yes	Cannot tell	No	Yes	High	Low
Williams (1999) [35]	Yes	Yes	Yes	Yes	Yes	Yes	Cannot tell	Yes	Yes	High	Low

Reference: Critical Appraisal Skills Program (CASP) 2014. CASP checklists are available at http://www.casp-uk.net/#!casp-tools-checklists/c18f8 [24].

The authors of the CASP tool request this note for every use: “The authors of this tool do not endorse this report, in whole or in part.”

**Table 4:** Systematic review results presented alphabetically by author.

Author/year/ country	Focus	Data Collection			Results
		Subjects	Data source	Methods	
Aiken et al. (2006) [27], United States	To document outcomes of a demonstration program	240 patients “seriously chronically ill” (of whom 62 chronic obstructive lung disease and 130 congestive heart failure)		Randomized controlled trial Outcomes were assessed every 3 months by telephone discussion	Case managed patients had higher self-care management, lower symptom distress, greater vitality, and more legal preparation for death

Back et al. (2005) [29], United States	To examine resource use during the last 60 days of life; as compared between palliative care cases, managed persons were compared to and controls.	Seniors dying of cancer, October 1, 2001 to Oct 31, 2002 (82 in case managed intervention group, 183 controls)	CHIPS database and some electronic medical record data.	Quantitative analysis	Case managed seniors were less likely to die in hospital and less use of acute care hospital resources was also evident, as compared to seniors who did not receive case managed care

Browne and Braun [41] (2001), United States	Determine impact of case management, by assessing caregiver burden and care recipient effects after this program was discontinued	118 frail seniors (functionally frail in at least two activities of daily living and two instrumental activities of daily living, still living at home who had formerly received case managed home-based care; however, only 55 were able to be interviewed, and the others were too ill or impaired) and 106 family caregivers were interviewed	Interviews	Qualitative and quantitative data analysis	After the cessation of a case management program for home-based elderly persons, half of the responding caregivers reported deterioration in their own health and an increase in their emotional fatigue. The remaining seniors indicated that the program had been critical for their support and safety. The death rate was higher after program cessation

Elwyn et al. (2008) [42], US	To gain insight into the reasons for case managed reduction in hospital utilization	Reports by 5 case managers of care of 121 frail elders (assessed by practice teams as at high future risk of unplanned admission to hospital)	Embedded in a larger study	Qualitative analysis	The reduction in hospital use was correlated with an increased quality of life from the case managed care

Head and Cantrell (2009) [36], US	Illustrate the integration of case management within managed care	Single case study, palliative care team interventions	EOL managed care records	Case study of interventions	Case management ensured patient centered care, and this care was also cost effective

Head et al. (2010) [31], United States	To study a pilot project that integrated case management in a managed care program	35 palliative care patients (advanced heart, lung, liver, or neurological disease, HIV (AIDS), renal failure, or advanced cancer)	Integrated case management data	Quantitative, descriptive-comparative study.	There was a decrease in symptom distress one month after the onset of services, and with growing satisfaction with the case management services over the first 3-month period

Long and Marshall (1999) [37], United States	Compare health services use between dying persons who were receiving case management services and those not receiving it	34 patients (in last month of life) in case managed intervention group, 43 controls	Demographic information and health records	Quantitative data analysis.	Case managed clients were more likely to be admitted to hospital, to have longer hospital stays and to have more outpatient visits. Health care costs in the last month of life were 60% higher for the case managed group than the regular-care group. Case managers were mainly client advocates, with their clients receiving assistance in accessing needed health services

MacDonald et al. (1994) [43], United Kingdom	Assess the activities of nurse case managers, as well as the acceptability and perceived effectiveness of a new program of case management for terminally ill persons	199 cancer patients with predicted life expectancy of 1 year	Hospital and community-based healthcare workers, bereaved family members	Mail survey (healthcare workers) and interviews (bereaved family members)	Despite an expected visit after hospital discharge, only 62% were visited at home and 71% were telephoned at home. 21% were never visited at home or telephoned at home. 51% of contacts lasted 5 minutes or less; 59% of health care workers had not heard of the coordinating service, 87% thought it was beneficial, and 70% of comments about it were positive. Relatives reported issues, such as not getting ordered equipment; (46%) did not know how to get help (34%), had difficulty contacting a health care professional (7%), and (35%) found EOL care not well coordinated. Families of persons who did not get case managed care reported similar care issues

Meier et al. (2004) [38], United States	Determine the effectiveness of delivering palliative care through case management programs (in South Carolina)	321 patients (152 terminally ill persons in case managed intervention group), 169 controls (5 nurse case managers in intervention group, 4 in control group)	Clients followed until death or case closure	Outcomes (Edmonton Symptom Assessment Scale)	Early data revealed that palliative case managers were empowered to identify patient distress and given responsibility to take action on it and that this involved an improved working relationship with physicians. The model thus appeared feasible, as it also improved patient and family satisfaction

Naylor et al. (1999) [28], United States	Randomized control trial to examine the effectiveness of a nurse practitioner case management program lasting 4 weeks after hospital discharge	262 hospital patients 65+ years at risk of adverse outcomes, (124 in intervention group received postdischarge case management), 138 controls received “usual care”	Hospital services use, functional status, depression assessment, and patient satisfaction data	Quantitative data analysis.	Over the six-month study period, the intervention group patients were less likely to be readmitted to hospital (30% versus 37%) and had fewer days in hospital (270 versus 760) and a shorter average hospital stay (7.5 versus 11 days) and half the health care costs. No differences in functional status, depression, or patient satisfaction were found

Nickel et al. (1996) [39], United States	Randomized controlled trial study to assess the effectiveness of nurse case management for quality of life among AIDS clients living at home	57 patients with AIDS referred for home care (29 intervention, 28 control)	Quality of wellbeing Scale	Quantitative data analysis.	Quality of wellbeing scores declined rapidly for both groups, reflecting both a decline in quality of life and impending or actual death. The usual care group mean scores were lower than the intervention group scores. Half were deceased at 6 months. Wellbeing differences between the groups were not statistically significant

Pfeifer et al. (2006) [40], United States	Describe the evolution of a palliative care management pilot program	56 palliative care recipients with advanced cancer	Descriptive summary		Patient responses were mostly extremely positive. Many relied on the care manager for advice and direction, but they were always encouraged to be self-managers in following medical orders and being compliant with treatment protocols

Seow et al. (2008) [30], United States	Evaluate a cancer case management pilot program focusing on palliative care	89 palliative care patients with cancer (69 in intervention group, 20 in control)		Quantitative, statistical comparison	75% of eligible patients enrolled in the case management program, and 59% of these had no hospital admissions compared to 15% of patients in the comparison group

Spencer and Battye (2001) [34], England	Identify needed initiatives in home palliative care for children with advanced cancer	Children with advanced cancer; assessment of work of 35 health care professionals	Individual interviews and group discussions	Qualitative data analysis.	Many different approaches to case management were evident, as multiagency collaboration and service delivery were needed. Needed improvements were identified as better communication and liaison between all professionals involved, clearer roles (especially with regard to who was the case manager or case team), 24-hour specialist support, faster access to some services, and continuity of nursing and respite care. Community nurses were supposed to provide home support, with specialist nurses providing advice and support when needed

Spettell et al. (2009), [32] United States	To evaluate the impact of case management on the use of hospice and acute health care services	Intervention group: 3,491 enrollees in US health plan with usual hospice benefits, 387 with expanded hospice benefits, and 447 Medicare Advantage members with hospice benefits; control group: matched on age, severity of illness, and diagnosis, but died before specialized hospice care became available	Retrospective cohort design, three intervention groups each matched to a historical control group		Hospice use increased for all groups who received case management, as compared to the respective control groups: 30.8% versus 71.7% or 27.9% versus 69.8%

Twyman and Libbus (1994) [33], United States	Assess impact of case management on the number of days in hospital over the last 6 months of life as compared to days for persons who did not receive case management	100 individuals who died of AIDS 1989–92 in case managed intervention group; 99 controls	Retrospective medical and hospital records	Quantitative data analysis.	No significant difference in inpatient days was found (13.7 days for the case managed individuals versus 15.3 days for controls). Although there was not a major difference in hospital use, this may have been because the case managers acted as advocates for their clients, with increased hospital use, an outcome of this advocacy.

Williams (1999) [35], Canada	Identify deficiencies in palliative home care and remedies	Interviews with 3 case managers in Niagara region about their work and gaps in care	Grounded theory interviews	Qualitative data analysis	A wide range of service gaps were identified, including community services for informal caregivers, specialist palliative care providers, and with an inequity in home care services and quality of services across the region. Lack of timely services and minimal notification of new clients were also issues. Numerous strategies were identified. More community services include home care, more internal communications, and networking.
